# Silica Gels Doped with Gold Nanoparticles: Preparation, Structure and Optical Properties

**DOI:** 10.3390/gels9080663

**Published:** 2023-08-17

**Authors:** Dimitar Shandurkov, Nina Danchova, Tony Spassov, Vesselin Petrov, Roumen Tsekov, Stoyan Gutzov

**Affiliations:** Faculty of Chemistry and Pharmacy, Sofia University St. Kliment Ohridski, J. Bourchier Blvd. 1, 1164 Sofia, Bulgaria; fhds@chem.uni-sofia.bg (D.S.); ndanchova@chem.uni-sofia.bg (N.D.); tspassov@chem.uni-sofia.bg (T.S.); v.petrov@chem.uni-sofia.bg (V.P.); tsekov@chem.uni-sofia.bg (R.T.)

**Keywords:** silica gels, gold nanoparticles (AuNPs), optical spectra, thermal conductivity, composite materials

## Abstract

A novel, one-pot sol–gel preparation scheme leading to reproducible incorporation of 20–40 nm sized gold nanoparticles (AuNPs) in SiO_2_ gels is developed based on in situ reduction during gelation using chloroauric acid and ascorbic acid. Variation in the preparation conditions affects the chemical composition, optical properties and size distribution of the AuNPs incorporated in the silica gels. Different organic dopants, i.e., oleic acid, acetic acid or dodecanethiol, are applied to modify the final composite material and to control the rate of reduction and growth of the AuNPs in the gels. The synthesized samples are characterized by UV/Vis/NIR spectroscopy, X-ray diffraction, transmission electron microscopy, thermal conductivity measurements and DTA/TG measurements. The optical properties of the obtained composites are explained using Mie theory. The incorporation of AuNPs leads to an increase in the thermal conductivity of the silica gels. The best process method in this contribution is the use of NaOH as a gelation catalyst and oleic acid as an organic modifier, leading to 20 nm AuNPs dispersed in the silica matrix.

## 1. Introduction

Incorporating gold nanoparticles (AuNPs) in inorganic gels can be a useful approach to creating new materials with unique optical, catalytic, thermal and electric properties with broad applications in various fields such as nanotechnology, biomedical engineering and electrochemistry [1,2,3,4].

There are different techniques to incorporate AuNPs in inorganic gels or glasses such as electrochemical deposition, laser ablation or sol–gel chemistry [1,2,3]. The sol–gel method, based on the hydrolysis and condensation of metal alkoxides, is one of the most used approaches to incorporate metal nanoparticles into inorganic oxides. Gold precursors are typically added in the form of gold salts, such as chloroauric acid (HauCl_4_·xH_2_O), and the reducing agent can be an organic or inorganic compound, such as sodium borohydride, citrate or ascorbic acid (L-AsAc) [5]. The AuNPs are embedded within the solid gel matrix and their size and distribution can be controlled by adjusting the synthesis parameters, such as the concentration of the gold precursor, the reducing agent and the pH.

A special direction in the incorporation of silver and gold nanoparticles into solid glass matrixes is the production of Au mirrors, thin films and glass decorations [6]. The optical properties of AuNPs are well described by Mie theory, where qualitative dependencies between surface plasmonic frequency and intensity, depending on particle size and the medium’s dielectric constant, present the possibility for a deeper description of composite materials on a molecular level [7].

Despite numerous investigations, there are many open questions concerning AuNP incorporation in solid matrixes, connected to the complex character of the processes in silica sol–gel matrix production [8,9]. For example, the use of organic modifiers as complex formation agents changes both the microstructure and the optical properties of the final sol–gel materials [10]. On the other hand, the complex nature of the silicate gels doped with gold nanoparticles allows one to vary the chemical composition of the doping agent in combination with the main parameters of the sol–gel process, as well as to protect the gold nanoparticles with a shell through additional additives.

While the preparation of tunable gold nanoparticles in solutions is relatively easy, their incorporation into gel matrixes is related to various physico-chemical problems related to the doping process, control of dispersity and AuNP agglomeration in the solid matrix [4,5,7]. The reproducible synthesis of inorganic and hybrid gels doped with gold nanoparticles is a prerequisite for the preparation of micro- and nano-powders with applications related to the functionality of gold nanoparticles.

The purpose of this study is to investigate how one-pot sol–gel preparation conditions affect the structure, optical properties, morphology and thermal conductivity of AuNP-doped silica gels (SiO_2_:AuNPs), starting from a tetraethyl orthosilicate solution (TEOS) and using ascorbic acid as a reducing agent. Moreover, the present study aims to demonstrate a novel, in situ doping method in which the reduction of the gold salt occurs during the gelation process. A similar method was successfully used for the preparation of hybrid optical materials containing lanthanide complexes in aerogels [11,12,13,14,15,16,17,18].

## 2. Results

**Figure 1:**
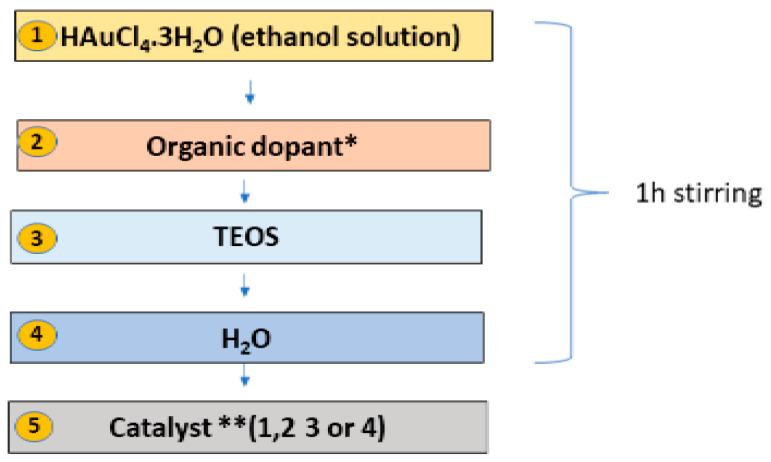
In situ one pot sol–gel preparation of SiO_2_:AuNPs. The catalysts used here are described as follows: **Catalyst 1**—NH_3_, H_2_O, EtOH; **Catalyst 2**—NH_3_, H_2_O, EtOH, L-AsAc; **Catalyst 3**—NaOH, H_2_O, EtOH; **Catalyst 4**—NaOH, H_2_O, EtOH, L-AsAc.

In this work, a specific in situ one-pot sol–gel procedure was developed for the preparation of SiO_2_:AuNPs, allowing control of the AuNP sizes dispersed in the silica matrix to between 10 nm and 40 nm. The synthesis started with tetraethyl orthosilicate (TEOS) using a gold salt solution (HAuCl_4_·3H_2_O) and ascorbic acid (L-AsAc) as a reducing agent. The sol–gel scheme used here is visualized in Figure 1. The samples were prepared with a 0.012 M HAuCl_4_.3H_2_O alcoholic solution. After stirring for 5 min, TEOS was added, followed by the addition of water, and the sol was homogenized for one hour. The molar ratio, n_Au_/n_TEOS_, used was 3.003 × 10^−4^ for all the samples in this work. An alkaline catalyst was added after the homogenization and hydrolysis of the sol, Table 1. After gelation, all gel samples were left in a sealed container for 24 h to age and which was then opened to dry under ambient conditions.

Different organic dopants were applied to modify the final composite material and to control the rate of reduction and growth of the AuNPs in the gels. Four types of catalysts were used and changed to modify AuNP formation (Table 1), designated as Catalysts 1–4. The catalysts added here act on both the gelation and the reduction of the gold ions (in situ formation) simultaneously. Finally, centimeter-scale, non-uniform bulk composite gels were produced, which were processed into micropowders.

Additionally, gold nanopowders were prepared by reduction of a 0.2 M solution of HAuCl·3H_2_O with a 0.2 M solution of L-AsAc, resulting in a gold precipitate with mean crystallite sizes of 35–40 nm, calculated from X-ray diffraction patterns [11].

**Figure 2:**
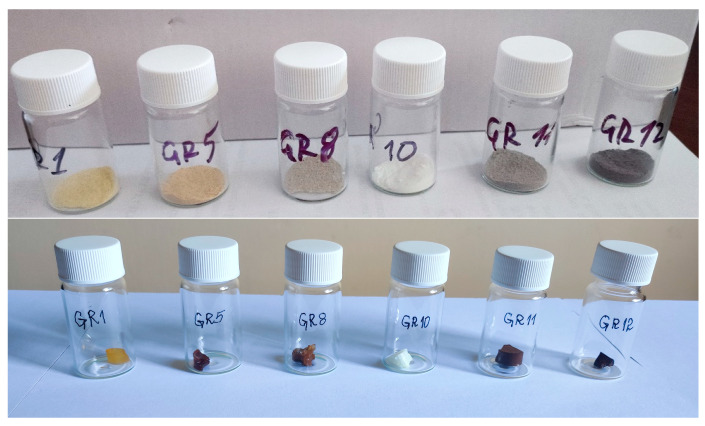
Photographs of powdered composite samples and centimeter-scale gels. The notation is the same as in Table 1. In the sample GR10, X-ray diffraction peaks from AuNPs were not detected.

Photographs of powdered samples and bulk gels of SiO_2_:AuNPs are shown in Figure 2.

The color of the powders (Figure 2) depends on the presence of AuNPs, organic residues and Au(NH_3_)_x_Cl_4-x_ complexes, as discussed in the next section. The colors are more saturated in the bulk gels. Yellow or white coloration can be attributed to the presence of HAuCl_4_·3H_2_O or the formation of Au(NH_3_)_x_Cl_4-x_ complexes (sample GR10) and red-brown gels contain AuNPs.

The formation of SiO_2_:AuNPs is a complex process, including the reduction and growth of AuNPs during gelation. From a physicochemical point of view, there are three processes producing AuNPs:

(i) Reduction of HAuCl_4_·3H_2_O with L-AsAc [12], which has been a well-known process for many years.
(ii) Presence of Au^0^ according to the Pourbaix diagram [13] under the specific preparation conditions applied here (pH > 7 and reduction potential 1.4 V), assuming [Au] less than 0.5%.
(iii) Reduction caused by active surface defects in silica. There is much spectroscopic evidence for such defects in glasses and silica gels, affecting the optical properties of these materials (blue emission, specific UV absorption and excitation spectra) [8,14,15].

In article [8], the authors argue that the reduction of Au(III) ions on silicate surfaces occurs on defects due to radicals. These defects are usually obtained when chemical bonds in the silicate skeleton are broken, and due to their high activity, they are relatively short lived. The third process would be significant when a solution of HAuCl_4_·3H_2_O is added to freshly ground silica or gel powders, as is the case in this study. The production rate of AuNPs, however, can be decreased by the presence of ammonia, leading to the formation of Au(NH_3_)_x_Cl_4-x_ polymer-like complexes with a UV/Vis absorption below 500 nm [16,17]. It follows that Catalyst 1 would promote Au(NH_3_)_x_Cl_4-x_ formation; Catalyst 2 would promote the formation of AuNPs under these conditions; Catalyst 3 would promote the “defect reduction on silica surfaces” leading to AuNPs; and Catalyst 4 would promote an improved formation of AuNPs in the presence of NaOH [13].

Moreover, organic modifiers oleic acid (OlAc), acetic acid (CH_3_COOH) or dodecanethiol (DDT) were added to the sol to protect/capsulate the produced nanoparticles. Details about the action of such modifiers are discussed in [18,19]. In solution, the formation and growth of AuNPs can be described by the Finke–Watzke model, which infers a fast reduction and a slow crystal growth [20,21]. Gelation, resulting in increasing viscosity, controls the incorporation of AuNPs in the gel. If the growth rate of AuNPs is too high compared to the gelation rate, the AuNP agglomerates become large and precipitate on the bottom of the plastic reaction container. If the reduction in the gels, however, is very slow, a partial reduction of HAuCl_4_·3H_2_O takes place on the walls of the PP container (Sample GR13, Table 1).

## 3. Discussion

### 3.1. Phase Composition and Microstructure of the Composites

**Figure 3:**
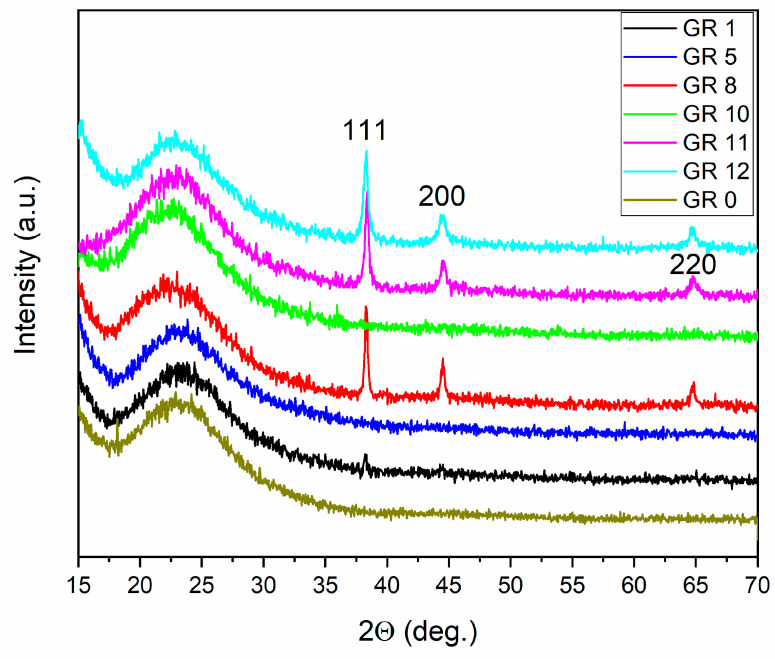
X-ray diffraction patterns of AuNPs containing composites. The hkl indexes of Au ICDD: 00-001-1172 are shown. The reference amorphous sample G0 does not contain AuNPs or organic modifiers.

A microstructural analysis was performed according to Au XRD peaks (ICDD: 00-001-1172). Peaks from Au_2_O_3_ or Au(OH)_3_ were not detected [22]. In samples GR13 and GR14 (Table 1), prepared with Catalyst 3 and Catalyst 4 (containing NaOH), traces from sodium silicate hydrate (Na_2_SiO_3_.6H_2_O) were found as well [23], Figure 3.

The mean particle diameter of the AuNPs incorporated in the samples was determined in the range of 20–40 nm. This value is close to that of microcrystalline gold precipitates (Au) reduced in a 0.2 M solution with L-AsAc, which is about 40 nm. For samples GR12 and GR14, where Catalyst 4 was used, the capping agent OlAc reduces the particle size in sample GR14 even further; an average particle size of 20 nm was measured. This observation gives a clear indication that the variation in the one-pot preparation conditions, especially the use of oleic acid, changes the sizes of the AuNPs incorporated in the silica matrix. In some samples, Au peaks could not be detected by X-ray diffraction because of the formation of amorphous gold complexes (discussed in Section 3.2).

The results of the X-ray diffraction analysis of microcrystalline Au powders and SiO_2_:AuNPs are summarized in Table 2. It is evident that both the mean crystallite size and Au lattice parameters depend on the preparation strategy, organic modifiers and gelation agent (NaOH or NH_3_). The mean lattice constant contraction detected in AuNPs embedded in silica matrixes and nanocrystalline Au precipitates (Table 2) is about 0.2%. This observation is quantitatively in agreement with the capillary effect of small particles [24,25].

**Figure 4:**
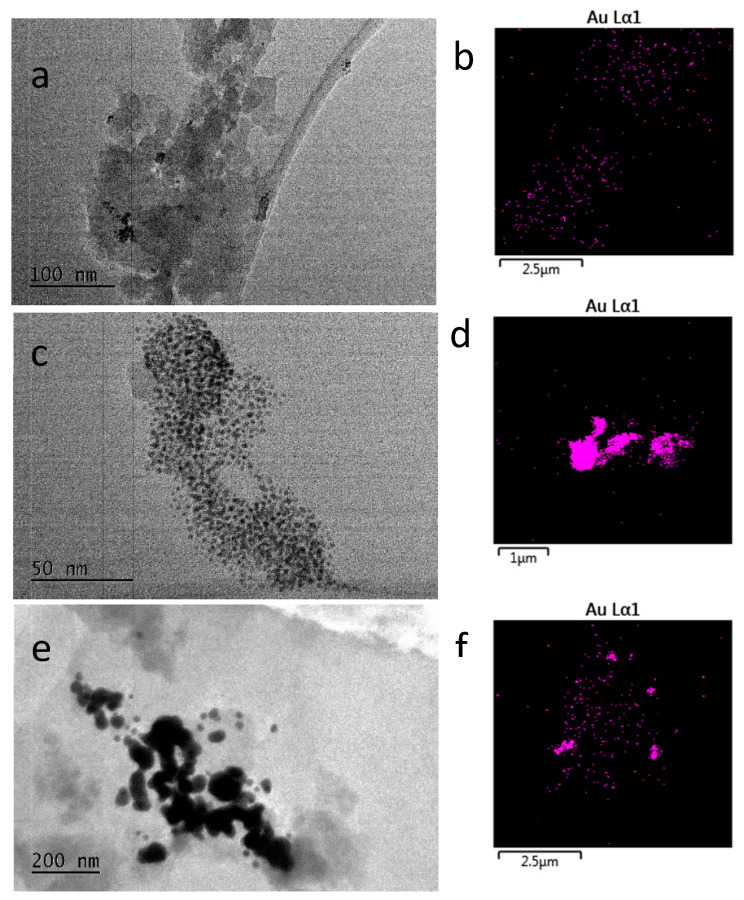
(**a**,**b**) TEM and EDAX images of sample GR1; (**c**,**d**) TEM and EDAX images of sample GR8; (**e**,**f**) TEM and EDAX images of sample GR12.

TEM/EDAX measurements unambiguously show the presence of AuNP agglomerates and individual nanoparticles of between 10 nm and 80 nm dispersed in the silica matrix, Figure 4. The TEM findings are supported by EDAX images, which give an idea of the gold particle distribution in the matrix. In sample GR1, where acetic acid was used as a modifying agent, the gold nanoparticles remain small (less than 15 nm in diameter) and rather spherical. This observation correlates with the morphology of zirconia gels obtained using acetic acid as a complex forming agent [10]. The particles are clustered, but not agglomerated. In sample GR8, however, where no modifier was used, the AuNPs remain smaller and seem to be more dispersed in the silica matrix.

The preparation conditions of sample GR12 led to the formation of bigger Au particles and their agglomeration. Thus, a comparison between the TEM/EDAX images shows that the preparation strategy effectively results in a diluted dispersion of AuNPs in the amorphous SiO_2_ matrix.

### 3.2. Thermal Conductivity

As expected, the presence of incorporated Au nanoparticles changed the thermal conductivity of the gel composites. The measured thermal conductivities of 0.08–0.1 W/m K of the composite are much higher than the conductivity of the silica matrix (0.068 W/m K), revealing a clear effect of the gold. Furthermore, dividing their difference by the molar fraction (0.0033) of gold in the composite gives a thermal conductivity of 5.455 W/m K, which is much lower than the conductivity of bulk gold (318 W/m K). Obviously, a small portion of gold, about 1.7%, could form percolation nets in the silica matrix.

The trend of increasing thermal conductivity due to AuNPs is in agreement with the results published in [26] for gold colloidal suspensions, where an increase in thermal conductivity in the range of 1–2% was reported. An increase in the thermal conductivity in TiO_2_ aerogels due to an optical band gap decrease was demonstrated recently [27].

**Figure 5:**
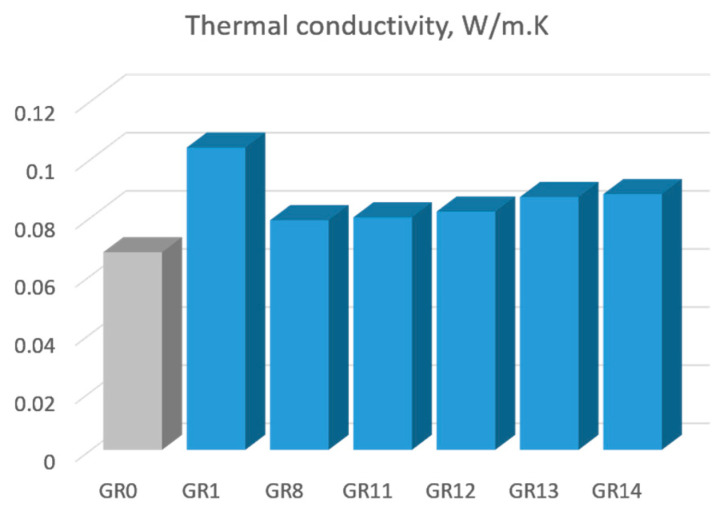
Thermal conductivity of silica gels containing AuNPs. The sample notation is the same as in Table 1. The sample GR0 is a pure silica gel.

The thermal conductivity of the samples, given in Table 2, is presented in Figure 5.

### 3.3. UV/Vis and NIR Reflectance Spectra

UV/Vis/NIR reflectance spectra of the composites prepared are displayed in Figure 5. The complex character of the optical properties of the as-prepared composite is clearly visible and three basic spectral features can be distinguished, leading to sample coloration.

Peaks below 500 nm are related to electronic transitions from organic additives or Au(NH_3_)_x_Cl_4-x_ complexes. It is well known that Au(NH_3_)_x_Cl_4-x_ and Au(OH)_x_Cl_4-x_ complexes have intense absorbances from ligand to metal charge transfer transitions in the range of 250–400 nm [28,29,30]. The occurrence of such peaks without a spectral feature from AuNPs indicates an incomplete reduction.

Peaks in the surface plasmonic resonance (SPR) of AuNPs, depending on their size, were detected in some samples between 500 nm and 600 nm according to the spectra; the size dependence of the plasmonic resonance of AuNPs [31,32] is shown in the inset of Figure 5 [31].

The NIR peaks are assigned as overtones of organic molecules and water. We can distinguish two types of peaks in the NIR region. The first one originates from the first and second overtones of molecular vibrations and is observed in the region of 1400–1800 nm, and the second, which is said to contain combinational vibrations, occurs between 2000 and 2400 nm [33].

All samples have peaks at about 1910 and 1410 nm which are designated to the first and second overtones, respectively, of water molecules and free −OH groups. The 1910 nm band is a combination of asymmetric stretching and bending of water molecules, which is usually found at 1940 nm but can shift towards lower wavelengths in the presence of alcohols. The samples containing oleic acid (GR3, GR4, GR7, GR8, GR13 and GR14) and the samples containing dodecanthiol (GR9 and GR10) have a doublet of peaks at 1725 and 1760 nm. These are ascribed to the first overtones of the C-H group bending mode in the organic molecule tail. The second overtone of this vibration is observed at 1210 nm with a much lower intensity than the first overtone. The first overtone of S-H is usually a weak band around 1970–1980 nm and is not present in the spectra of samples GR9 and GR10. These samples also display a peak at 2345 nm, which is most likely a combinational vibration. All samples have four peaks situated at 2210, 2250, 2300 and 2440 nm, which are combinational vibrations. The bands at 2210, 2250 and 2440 nm are said to originate from the combination of the −OH stretch with one of the SiO_2_ fundamental vibrations.

No peaks originating from acetic acid were found which means that the acetic acid has evaporated from the samples and, at most, trace amounts of it can be found in the samples [33].

**Figure 6:**
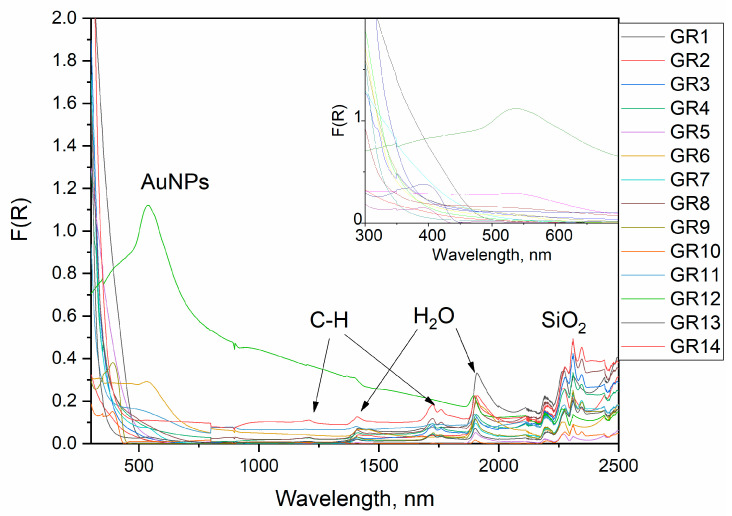
UV/Vis/NIR reflectance spectra of the composites. The spectral region of the surface plasmonic resonance is given as an inset. The origin of the NIR and UV peaks is shown.

In some cases, the typical surface plasmonic peaks of AuNPs at 500–580 nm are not visible in the reflectance spectra or appear as a shoulder only. Similar differential spectra measurements are presented in Figure 6, where the pure sol–gel silica powder matrix is used as a reference. Here, the increased F (R) values compared to that of the pure SiO_2_ matrix suggest a light scattering due to imperfections or nanoparticles. Typical Au peaks, however, are visible in the X-ray diffraction diagrams of the respective samples (GR1). In the second derivative of the diffuse reflectance spectra, only weak evidence in the region of surface plasmonic resonance is detected as follows: sample GR1 at 520–540 nm and samples GR9, GR4 and GR2 at 530–560 nm. The most probable explanation of the low intensity of the surface plasmonic transition here could be the formation of a non-transparent shell around the AuNPs.

**Figure 7:**
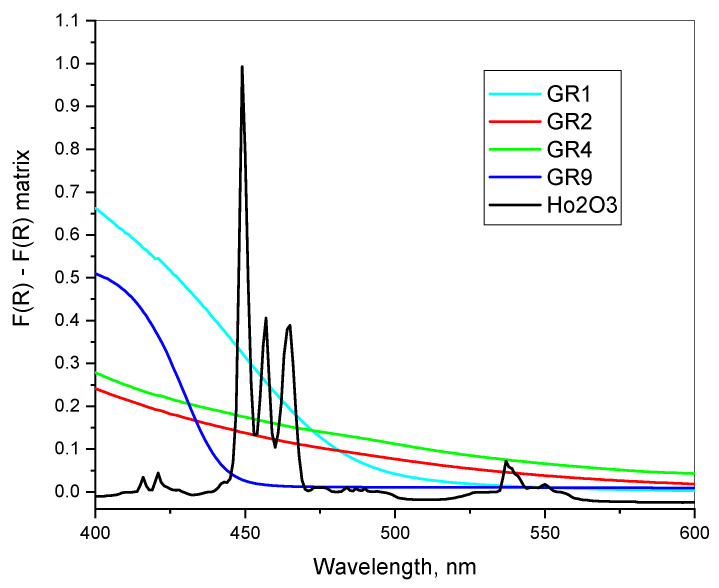
Differential UV/Vis reflectance spectra of SiO_2_:AuNPs using pure sol–gel SiO_2_ powders as a reference (GR0). The spectrum of Ho_2_O_3_ powders (Sigma-Aldrich, Darmstadt, Germany, 99.9+) is given for comparison.

The results in Figure 7 suggest a possible surface modification of AuNPs with organic modifiers. This suggestion is also supported by the calculation of the high frequency dielectric permittivity, $\varepsilon_{m}$, in the next section.

### 3.4. Spectra Evaluation According to Mie Theory

The dynamics of electron gas undulations in metals generated by an external oscillatory electric field are traditionally described by the Drude model [34]. Accordingly, the wavenumber dependence of the dielectric permittivity of AuNPs acquires the well-known form:

(1) $$\varepsilon (\tilde{\nu })=1-\frac{{\tilde{\nu }}_{sp}^{2}}{{\tilde{\nu }}^{2}+i\tilde{\nu }\gamma }$$

where the surface ${\tilde{v}}_{sp}$ plasmon wavenumber is related to the Langmuir ${\tilde{\nu }}_{p}$ plasma wavenumber via the expression:

(2) $${\tilde{\nu }}_{sp}^{2}={\tilde{\nu }}_{p}^{2}/(\varepsilon_{m}+1)$$

Here, $\varepsilon_{m}$ is the dielectric constant of the medium surrounding the nanoparticles. For bulk gold, ${\tilde{v}}_{p}=72,000\;{\mathrm{cm}}^{-1}$ and the high-frequency dielectric constant of water is about $\varepsilon_{m}=1.77$. The collision wavenumber $\gamma =v_{F}/cl$ is equal to the ratio between the relative Fermi velocity in bulk gold $v_{F}/c=0.0046$ and the mean-free path of the electron gas. It accounts for collisions of free electrons with the ions of the metal lattice. The mean-free path in bulk gold is about 38 nm [35], but it could be essentially reduced in smaller nanoparticles by their size and shape due to a larger Knudsen number.

According to Mie theory [34], the intensity distribution $I(\tilde{\nu })$ of the absorption spectrum of gold nanoparticles is solely determined by the dielectric permittivity of AuNPs given by Equation [34] and $\varepsilon_{m}$:

(3) I(ν˜)∼εmεIm(εRe+2εm)2+εIm2∼ν˜maxγ(ν˜−ν˜max)2+(γ/2)2

Obviously, the full half width Δ of the Lorentzian spectrum (3) is equal to the collision wavenumber γ. Hence, one can easily estimate the mean-free path l=0.0046/Δ from the experimental values of Δ in Table 3. If the mean-free path is smaller than 38 nm, the average size of the nanoparticles should be comparable to l.

The calculated values of l are in good agreement with the mean particle sizes of AuNPs extracted from X-ray diffraction patterns (see Table 2) and with the TEM observations, showing single AuNPs with sizes lower than 40 nm.

Equation (2) also predicts the resonance condition εRe=−2εm, which defines the maximum wavenumber of the Lorentzian peak via:

(4) $${\tilde{\nu }}_{\max}^{2}={\tilde{\nu }}_{sp}^{2}/(2\varepsilon_{m}+1)-\gamma^{2}$$

As is seen, larger nanoparticles possess lower collision wavenumbers, which correspond to a blue shift of the peak [4]. Using this expression, one can calculate the dielectric constant of the surrounding medium of AuNPs from the spectral characteristics:

(5) $$\varepsilon_{m}=[\sqrt{1+8{\tilde{\nu }}_{p}^{2}/({\tilde{\nu }}_{\max}^{2}+\Delta^{2})}-3]/4$$

**Figure 8:**
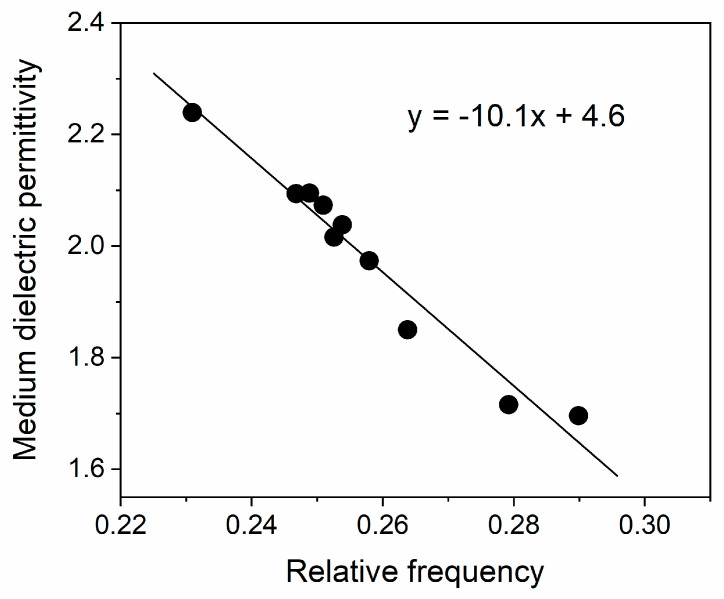
Dependence of the medium dielectric permittivity $\varepsilon_{m}$ on the ratio ${\tilde{\nu }}_{\max}/{\tilde{\nu }}_{p}$ between the maximum plasmonic and the bulk plasma wavenumbers.

The calculated values from Equation (5) are also presented in Table 3. In Figure 8, the dependence of $\varepsilon_{m}$ on the relative wavenumber ${\tilde{\nu }}_{\max}/{\tilde{\nu }}_{p}$ is plotted.

As expected, the dielectric permittivity decreases with an increase in the frequency and the extrapolated static dielectric constant ($\varepsilon_{m}(0)=4.6$) is close to the upper limit of the literature value for silica.

Equation (1) predicts the occurrence of AuNP absorption peaks at wavelengths lower than 500 nm, depending on the value of $\varepsilon_{m}$. Such peaks, however, overlap with the electronic transitions of organic modifiers used here and of Au(NH_3_)_x_Cl_4-x_ complexes.

### 3.5. Thermal Stability of the Composite Gels

**Figure 9:**
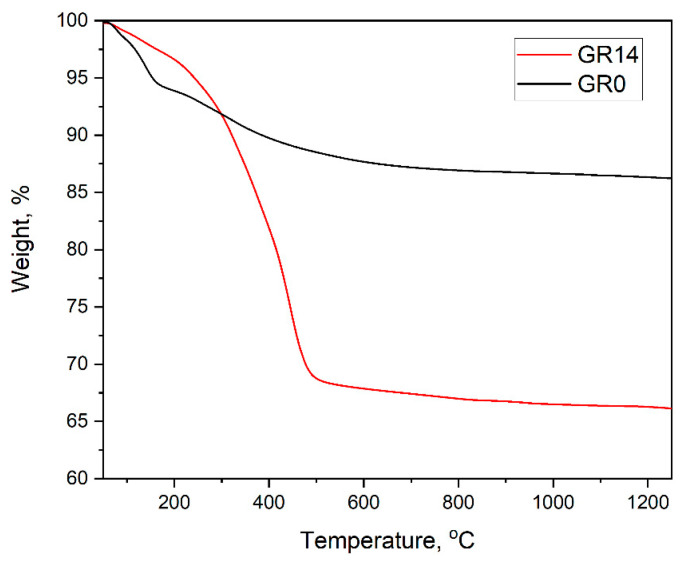
TGA plots of samples G0 (silica gel without AuNPs) and Gr14 (silica gel with 20 nm AuNPs; oleic acid used as organic modifier). The sample notation is the same as in Table 1.

The TGA plots in Figure 9 reveal the similar thermal stability of the two gel samples, typical for hybrid silica sol–gel micropowders. The silica gel micropowders containing AuNPs are stable up to 60 °C, which is suitable for possible drug carrier applications. Processes typical of sol–gel powders take place, i.e., dehydration (physically bonded water) followed by decomposition (chemically bonded water), organic modifier decomposition and their chemical reaction with the silica matrix, at temperatures above 200 °C [17,27,36].

The curves of silica gel with AuNPs and pure silica gels differ because of organic modifier (oleic acid) additions, which additionally results in about a 30% mass loss. The overall mass loss of G0 after heating is about 15%, in agreement with data on the thermal stability of silica gels [10,14]. The samples are stable above 450 °C, up to the melting temperature of gold. The TGA findings are well supported by DTA measurements.

## 4. Materials and Methods

The following chemicals were used for the syntheses of the composite materials: absolute ethanol (abs EtOH), tetraethyl orthosilicate (TEOS), tetrachloroauric acid trihydrate (HAuCl_4_.3H_2_O), glacial acetic acid (CH_3_COOH), oleic acid (OA), 1-dodecanethiol (DDT), ammonia solution (NH_3_), sodium hydroxide (NaOH), L-ascorbic acid (L-AsAc) and distilled water. All reagents, apart from oleic acid, were of analytical grade and were used without further purification. The oleic acid and the ammonia solution were provided by a local supplier and all the other chemicals were supplied by Sigma-Aldrich. All syntheses were carried out in sealed plastic PP containers. The sols were stirred with Teflon-coated magnetic bars.

UV/Vis/NIR powder reflectance spectra were measured on an Agilent Cary 5000 spectrophotometer with a “Praying mantis” sample holder using Spectralon^®^ as a reference between 200 nm and 2500 nm. The Kubelka–Munk function $F(R)={\left(1-R\right)}^{2}/2R$ was calculated from the diffuse reflectance R of all samples. The spectral features of the AuNPs peaks were analyzed as follows: second derivatives to obtain spectral maxima, followed by a single Lorentzian fit to extract peak position, full width at half maximum (FWHM) and integrated absorption intensities (I). The spectral features of AuNPs were interpreted according to Mie theory. The investigated samples show a significant diffuse reflection from the SiO_2_ powder matrix together with that of the AuNPs. Reference reflectance measurements were taken on a PE Lambda 35 spectrometer with a Spectralon^®^-coated integrating sphere.

As a standard for all reflection measurements, Ho_2_O_3_ powder was used, and all peaks and relative intensities of the Ho(III) f–f transitions at 300–2000 nm are in agreement with spectral data published in [37].

The structure and microstructure of the samples were characterized by X-ray diffraction with Cu-Kα radiation (Siemens D 500 diffractometer, at a step of 0.05 Θ and counting time of 4 s/step). The mean crystallite sizes were calculated using Scherrer’s equation for the most intense Au peak (111). The error of the lattice constant determination is about 0.005 Å.

TEM/EDAX investigations were performed using a JEOL 200 transmission electron microscope and for the SEM observations, a JEOL JSM5510 was used.

The thermal conductivities were measured on a C-Therm MTPS (Modified Transient Plane Source) TCi Thermal Conductivity Analyzer equipped with a powder and liquid sample holder. The error of the thermal conductivity determination of the gel powders is about 0.0002 W/m·K; R^2 = 0.999. The powders were measured under identical measurement conditions.

To study the thermal stability of the gels and gain additional information on their structure, thermogravimetric analysis (TA SDT 650) was applied under a nitrogen atmosphere (200 mL/min) at a 10 °C/min scanning rate in the temperature range of 50–1200 °C.

## 5. Conclusions

Silica gel composites containing gold nanoparticles (AuNPs) are obtained using a novel in situ one-pot sol–gel preparation method, starting from TEOS, HAuCl4 and L-ascorbic acid as a reduction agent. The structure, chemical composition and optical properties of the gels were found to depend on the complex catalyst used in the sol–gel process. Variation in the preparation conditions affects the chemical composition, optical properties and thermal conductivity of the composite silica gels. The sizes of the AuNPs are reduced by the presence of oleic acid from 40 nm to 20 nm. Complex-forming substances such as NH_3_ and DDT inhibit the formation of AuNPs, while NaOH increases the amount of AuNPs in the silica gels. The best process method in this contribution includes the use of NaOH as a gelation catalyst and oleic acid as an organic modifier.

Using a theoretical approach based on Mie theory, it was established that the photophysical properties of the obtained composites are tunable due to AuNP surface plasmonic resonance.

A significant increase in the thermal conductivity of about 10–15% is detected as a result of AuNP incorporation in silica gels.

## Figures and Tables

**Table 1:** Chemical composition, organic modifiers and catalysts used in the preparation of the samples.

Sample	Catalyst Number	Organic Dopant	Mol ratio n_TEOS_/n_Organic dopand_	Organic Dopants (mM)	n_NH3_ (mM)	n_NaOH_ (mM)	n _L-AsAc_ (mM)
GR1	1	AAc	0.855	26.2	1	-	-
GR2	2	AAc	0.855	26.2	1	-	4.26 × 10^−2^
GR3	1	AAc and OlAc	0.855 9.825	26.2 and 2.28	1	-	-
GR4	2	AAc and OlAc	0.855 9.825	26.2 and 2.28	1	-	4.26 × 10^−2^
GR5	1	-	-	-	1	-	-
GR6	2	-	-	-	1	-	4.26 × 10^−2^
GR7	1	OlAc	9.825	2.28	1	-	-
GR8	2	OlAc	9.825	2.28	1	-	4.26 × 10^−2^
GR9	1	DDT	9.868	2.28	1	-	-
GR10	2	DDT	9.868	2.28	1	-	4.26 × 10^−2^
GR11	3	-	-	-	-	1	-
GR12	4	-	-	-	-	1	4.26 × 10^−2^
GR13	3	OlAc	9.825	2.28	-	1	-
GR14	4	OlAc	9.825	2.28	-	1	4.26× 10^−2^

**Table 2:** X-ray diffraction data of SiO_2_:AuNPs and precipitated Au nanopowders (*—traces).

Sample	Phases	D (nm)	Au Lattice Constant (Å)
GR1	SiO_2_:AuNPs	40	4.071
GR11	SiO_2_:AuNPs	30	4.059
GR8	SiO_2_:AuNPs	40	4.064
GR12	SiO_2_:AuNPs	25	4.068
GR13	SiO_2_:AuNPs; Na_2_SiO_3_ *	35	4.063
GR14	SiO_2_:AuNPs; Na_2_SiO_3_ *	20	4.068
GR0	SiO_2_, amorph	-	-
Au precipitates	Au	40	4.050

**Table 3:** Spectroscopic quantities of SiO_2_:AuNPs according to Mie theory. All the spectral data in Table 3 were obtained under the same spectroscopic conditions.

Sample	${\tilde{\nu }}_{\max}$ (cm^−1^)	Δ (cm^−1^)	l (nm)	$\varepsilon_{m}$
GR3	20.873	1472	31	1.7
GR5	18.068	1147	40	2.1
GR6	17.768	2699	17	2.1
GR7	18.571	2734	17	2.0
GR8	20.104	5151	9	1.7
GR11	18.993	5134	9	1.8
GR12	18.184	3307	14	2.0
GR13	18.278	1448	32	2.0

## Sample Availability

Samples of the compounds are available from the authors.
